# Nutrition knowledge among adolescents who participate in sports: a cross-sectional study from Vancouver, Canada

**DOI:** 10.3389/fnut.2026.1898385

**Published:** 2026-07-29

**Authors:** Enhui Evelyn Zhang, Alysha L. Deslippe, James McKendry, Tamara R. Cohen

**Affiliations:** 1 Food, Nutrition and Health, Faculty of Land and Food Systems, University of British Columbia, Vancouver, BC, Canada; 2 Department of Health, Kinesiology and Applied Physiology, Concordia University, Montreal, QC, Canada; 3 BC Children’s Hospital Research Institute, Healthy Starts, BC Children’s Hospital, Vancouver, BC, Canada

**Keywords:** adolescent athletes, Canada, dietary behaviour, nutrition education, sports nutrition knowledge

## Abstract

**Background:**

Adequate nutrition, defined as meeting energy and nutrient requirements to support sport-related needs, metabolic function, and growth, is critical for adolescent (14–18 years) athletic performance and health. Many adolescents do not meet current nutrition recommendations and report poor awareness of their nutrient needs (i.e., nutrition knowledge), though trends may differ between male and female adolescents. Most evidence is derived from the United States, with limited data from Canadian populations. The aim of this study was to assess sport nutrition knowledge among male and female adolescents participating in organized sport in the Greater Vancouver area (Canada).

**Methods:**

Adolescents participating in organized sport completed an online questionnaire that collected information on their sport participation, nutrition support and nutrition knowledge using the validated Abridged Nutrition for Sport Knowledge Questionnaire (A-NSKQ; 35 items). Descriptive statistics were generated, and independent samples t-tests assessed sex differences. Multivariable linear regression models were used to compare nutrition knowledge scores between sexes while adjusting for age and sports participation characteristics.

**Results:**

Fifty-one adolescents participated (*n* = 31 females). Few adolescents reported access to a team dietitian (6.1%) or structured nutrition education (26.5%). The mean total A-NSKQ score was 16.49 ± 4.38 (47.1% correct), indicating poor nutrition knowledge. Mean general and sport nutrition scores were 6.14 ± 1.92 (of 11) and 10.35 ± 3.08 (of 24), respectively. No significant sex differences were observed in total, general, or sport nutrition knowledge (all *p* > 0.05).

**Conclusion:**

Adolescent athletes in this Greater Vancouver sample demonstrated poor nutrition knowledge, with no differences between sexes. Limited access to dietitians and structured education suggests a need for improved sport nutrition education to support health and athletic development.

## Introduction

Adequate nutrition, defined as consuming sufficient energy and nutrients to support sport-related training, metabolic function, and growth, is fundamental to athletic performance and long-term health (1, 2). During adolescence, energy and nutrient demands are compounded by rapid growth and development associated with puberty, resulting in increased requirements. Inadequate intake during this period has been associated with negative impacts on growth potential, long-term bone health, and athletic performance (1, 3).

Adolescent athletes represent a heterogeneous population. In many contexts, this term includes adolescents participating across a spectrum of recreational to competitive sport environments. In general, nutritional requirements vary according to the training volume, and competition demands of the sport they participate in (4). Adolescents with higher training loads, including greater training frequency, duration, and intensity, may have higher energy demands than those participating at a more recreational level (5). Biological sex is also an important consideration during adolescence as nutritional requirements (e.g., iron) and metabolic rate differ between the sexes due to variations in growth, metabolic functioning, and body composition (6). These physiological differences underscore the importance of examining nutrition knowledge by sex, as knowledge gaps may have distinct implications for male and female adolescents’ health and athletic performance during this critical period of growth and development (1, 3).

Nutrition knowledge has been identified as an important, modifiable factor influencing dietary behaviours among athletes (7–9). Greater nutrition knowledge has been associated with improved dietary intake and better alignment with sport nutrition recommendations, such as healthier food choices, improved dietary quality, and more appropriate nutrition practices surrounding training and recovery (7–9). Importantly, evidence from a two-year sport nutrition education intervention among 217 high school soccer players suggests that improvements in nutrition knowledge can lead to improved sport nutrition knowledge, healthier dietary behaviours, and more positive attitudes toward healthy eating and sport nutrition among adolescent athletes (10). In addition, a systematic review has highlighted the importance of modifiable behavioural factors in athletes for improving nutrition behaviours, including knowledge in influencing dietary intake (9). Previous studies have reported sex differences in nutrition knowledge, attitudes and dietary behaviours among adolescent athletes, although findings have been inconsistent. These differences may reflect variations in physiological requirements, body composition, sport participation patterns, and nutrition-related attitudes and experiences (5, 6, 8, 11, 12). Given that many adolescent athletes are beginning to make more independent dietary decisions (13), adequate foundational nutrition knowledge at this life stage may play an important role in guiding these decisions (14).

Adolescents may obtain nutrition information through formal education and informal sources, including coaches, parents, peers, and online platforms (6, 8, 15, 16). While these sources can increase exposure to nutrition-related information, the quality and accuracy of available content vary substantially. For example, athletes frequently report relying on coaches, teammates, and social media as nutrition information sources, despite evidence that online nutrition-related information is often inconsistent, incomplete, or not evidence-based, potentially contributing to confusion or suboptimal dietary practices (8, 15). Family and team environments may also influence how nutrition information is interpreted and applied in daily life (16).

Existing literature consistently reports poor general and sport nutrition knowledge among adolescent athletes. Notably, these studies have been conducted outside of Canada, resulting in a lack of Canadian-specific evidence investigating adolescent athletes’ nutrition knowledge (3, 17, 18). Instead, a single study conducted with Canadian adolescent athletes focused on supplement-related knowledge, rather than overall sport nutrition knowledge (19). This lack of Canadian data restricts the development of targeted, contextually relevant nutrition education strategies for adolescent athletes. Thus, given the established links between nutrition knowledge and dietary behaviours, an improved understanding of nutrition knowledge among adolescent athletes in Canada is warranted. To help address this critical gap, the aim of this study was to assess sport nutrition knowledge among adolescents aged 14–18 years participating in organized sport in the Greater Vancouver area, and to examine potential sex differences in nutrition knowledge.

## Methodology

### Study design and ethics approval

This cross-sectional study collected data using REDCap (Research Electronic Data Capture), a secure web-based platform (20, 21). Ethics was approved through the University of British Columbia Research Ethics Board (Vancouver, BC, Canada; H25-01335).

### Participants and recruitment

In the present study, “athletes” were defined as adolescents participating in organized sport, including both competitive and recreational contexts with structured training beyond general physical education. In this context, our definition of athlete reflects a broad, heterogeneous adolescent sport-participating population rather than exclusively trained or elite athletes. Eligibility was based on self-reported participation in organized sport, including reported years of sport participation and weekly training hours. Participants were primarily recruited from secondary schools across the Greater Vancouver area (including the Vancouver, Burnaby, North Shore, Surrey, and Tri-Cities school districts, as well as independent schools). All adolescents (14 to 18 years) residing in the Greater Vancouver area were eligible if they were actively participating in at least one organized sport at the time of the survey. Recruitment occurred between January and February 2025 using posters, in-person outreach at a BC Children’s Hospital Research Institute event (Discovery Days in Health Science), and word-of-mouth. Snowball sampling was also used.

### Study procedure

Following informed online consent, adolescents completed a one-time anonymous survey using REDCap (20, 21) accessed via a scannable QR code on recruitment posters. Under the approved study protocol, the research ethics board approved and permitted adolescents between the ages of 14–18 years to provide their own electronic informed consent prior to participation. The ethics board did not require guardian consent to participate in this study. After survey completion, participants could optionally enter a draw for a $10 CAD e-gift card by leaving their email on a separate form to maintain anonymity.

### Measures

Nutrition knowledge was assessed using the validated 35-item Abridged Nutrition for Sport Knowledge Questionnaire (A-NSKQ) (22, 23), which includes general nutrition knowledge (11 items; examples: Q3: “Do you think cheddar cheese is high or low in fat?”; Q6: “The body has a limited ability to use protein for muscle protein synthesis”) and sport nutrition knowledge (24 items; examples: Q12: “Do you think 1 medium banana has enough carbohydrate for recovery from intense exercise?”; Q17: “Eating more protein is the most important dietary change if you want to have more muscle”). All items were presented using structured response options depending on question format (e.g., agree/disagree/not sure or enough/not enough/not sure). Correct responses were awarded 1 point, while incorrect and “not sure” responses received 0 points, therefore highest possible score was 35. Scores were calculated according to established A-NSKQ scoring procedures, with higher scores indicating greater nutrition knowledge. Total and subscale scores were calculated as raw scores and expressed as percentage correct. Based on established thresholds, scores were categorized as poor (0–49%), average (50–65%), good (66–75%), or excellent (76–100%) nutrition knowledge (22).

### Additional measures

Additional variables collected included demographics (age, sex at birth, gender identity, ethnicity) and sport-related characteristics (years of sport participation, training volume, competition frequency, and access to coaching or nutrition professionals).

### Statistical analysis

Descriptive statistics were conducted using RStudio (version 2025.x; RStudio, PBC, Boston, MA, USA) with R statistical software (version 4.5.x; R Foundation for Statistical Computing, Vienna, Austria). All variables were categorical and were reported as frequencies and percentages. Participants reported both sex at birth and gender identity; however, all sex-based analyses were conducted using self-reported sex at birth. Independent samples *t*-tests were used to compare mean nutrition knowledge scores between males and females for total, general, and sport nutrition knowledge. To adjust for potential confounders, multivariable linear regression models were used. Due to the small sample size (*n* = 51), a primary adjusted model controlled only for age, while an exploratory model further adjusted for sports participation characteristics (years playing main sport, training hours per week, and competitions per month). Data were assessed for normality and homogeneity of variance prior to analysis, and no data transformation was required. Statistical significance was set at *p* < 0.05. Item-level response patterns for the A-NSKQ were also examined descriptively by calculating the percentage of correct responses for each questionnaire item.

## Results

### Demographic and sport participation characteristics

Demographic and sport participation characteristics are presented (Table 1). A total of 70 adolescents initiated the survey, of whom 51 completed all A-NSKQ items and relevant demographic questions and were included in the final analysis. The majority of participants were female (60.8%) and 16 years of age (45.1%). More than half of participants identified as East Asian (52.9%), followed by European or White (35.3%) and South Asian (15.7%).

**Table 1:** Adolescent demographic and sport participation characteristics.

	Total sample(*n* = 51)	Males (*n* = 20)	Females (*n* = 31)
Age (years) [*n* (%)]			
14	1 (2.0)	1 (4.8)	0 (0.0)
15	9 (17.6)	3 (14.3)	6 (19.4)
16	23 (45.1)	5 (23.8)	18 (58.1)
17	9 (17.6)	2 (9.5)	7 (22.6)
18	9 (17.6)	9 (42.9)	0 (0.0)
Gender identity [*n* (%)]			
Boy	21 (41.2)	21 (100)	0 (0)
Girl	29 (56.9)	0 (0)	29 (93.5)
Prefer not to say	1 (2.0)	0 (0)	1 (3.2)
Ethnicity^†^ [*n* (%)]			
African	1 (2.0)	0 (0.0)	1 (3.2)
East Asian	27 (52.9)	11 (52.4)	16 (51.6)
South Asian	8 (15.7)	4 (19.0)	4 (12.9)
Southeast Asian	3 (5.9)	1 (4.8)	2 (6.5)
European/White	18 (35.3)	8 (38.1)	10 (32.3)
Middle Eastern	2 (3.9)	1 (4.8)	1 (3.2)
Parent/caregiver background in health, nutrition, or sport			
Yes	20 (39.2)	9 (42.9)	11 (35.5)
No	31 (60.8)	12 (57.1)	20 (64.5)
Sport participation characteristics			
Years playing (mean ± SD)	4.3 ± 2.3	4.5 ± 2.4	4.2 ± 2.2
Training hours/week (mean ± SD)	5.3 ± 3.4	5.9 ± 3.7	4.9 ± 3.2
Competitions/month (mean ± SD)	3.3 ± 1.7	3.6 ± 1.9	3.2 ± 1.6
Have a coach	35 (70.0)	16 (76.2)	19 (61.3)
Access to a team nutritionist/dietitian	3 (6.5)	1 (4.8)	2 (6.5)
Access to nutrition education through sport involvement	13 (26.5)	6 (28.6)	7 (22.6)

Values are presented as *n* (%) or mean ± SD. Percentages are based on the final analytic sample (*n* = 51) and may not total 100 due to rounding.

^†^Ethnicity was assessed as a multi-select variable, meaning adolescents could select more than one category; therefore, percentages do not sum to 100%.

Participants represented a variety of sports, most commonly badminton (*n* = 10), basketball (*n* = 9), volleyball (*n* = 6), and track and field/running (*n* = 6). Overall, 54% of participants were involved in individual sports, while 46% participated in team sports. In terms of level of participation, most adolescents competed at the club (31.3%) or school team level (27.1%), with fewer participating recreationally (16.7%), or at provincial (10.4%) and national (8.3%) levels.

Seventy percent of athletes indicated that they participated in a sport with a coach; those without a coach were primarily participating in structured recreational or community-based sport environments (e.g., the badminton club at the University of British Columbia). Few athletes reported having access to a team nutritionist or dietitian (6.1%), and sport-provided nutrition education opportunities (26.5%) (Table 1). In addition, 39.2% of adolescents reported that their parents or caregivers had a background in health, nutrition, or sports, and parents/family were the most reported source of nutrition information (39.2%).

### Overall nutrition knowledge scores

Nutrition knowledge scores are presented in Table 2. The mean general nutrition knowledge score was 6.14 ± 1.92 out of 11, and the mean sport nutrition knowledge score was 10.35 ± 3.08 out of 24. The mean total A-NSKQ score was 16.49 ± 4.38 out of 35, corresponding to a score of 47.1% correct responses. Based on established classification thresholds, this indicates poor nutrition knowledge (<50% correct). Unadjusted independent samples t-tests showed no significant sex differences in total A-NSKQ scores (male: 16.35 ± 4.37; female: 16.58 ± 4.46; *p* = 0.857), general nutrition knowledge (male: 5.75 ± 1.94; female: 6.39 ± 1.89; *p* = 0.251), or sport nutrition knowledge (male: 10.60 ± 2.89; female: 10.19 ± 3.23; *p* = 0.650) scores (Table 2). Furthermore, these sex differences remained statistically non-significant in primary multivariable linear regression models adjusting for age (total *p* = 0.706; general nutrition *p* = 0.482; sport nutrition *p* = 0.331), as well as in exploratory models additionally controlling for sports participation characteristics (all *p* > 0.05).

**Table 2:** Nutrition knowledge scores (*n* = 51).

Outcome	Items	Possible score range	Mean (SD)	Male mean (SD)	Female mean (SD)	Unadjusted *p*^a^	Age adjusted *p*^b^	Age + sport adjusted *p*^c^
General nutrition knowledge	11	0–11	6.14 (1.92)	5.75 (1.94)	6.39 (1.89)	0.251	0.482	0.402
Sport nutrition knowledge	24	0–24	10.35 (3.08)	10.60 (2.89)	10.19 (3.23)	0.650	0.331	0.585
Total A-NSKQ score	35	0–35	16.49 (4.38)	16.35 (4.37)	16.58 (4.46)	0.857	0.706	0.995

A-NSKQ, Abridged Nutrition for Sport Knowledge Questionnaire.

a Calculated using unadjusted independent-samples t-tests.

b Calculated using multivariable linear regression adjusting for age.

c Calculated using exploratory multivariable linear regression adjusting for age, years playing main sport, training hours/week, and competitions/month.

### Item-level performance on the A-NSKQ

Item-level performance scores from the A-NSKQ demonstrate substantial variability in correct responses across the 35 questions (Supplementary Table S1). Within the general nutrition knowledge section, the highest proportion of correct responses was observed for identifying cheddar cheese as high in fat (86.3%) and recognizing the importance of hydration (80.4%).

In contrast, knowledge of sport-specific nutrition recommendations was limited; only 7.8% of adolescents correctly identified the recommended post-exercise protein intake, and 5.9% recognized that not all supplements are supported by strong scientific evidence for performance enhancement. Relatively low accuracy (<50%) was also reported for questions related to recovery nutrition and micronutrient functions. Forty percent correctly identified appropriate carbohydrate intake during prolonged exercise and 60.8% correctly recognized the role of carbohydrates in maintaining blood glucose during exercise. When it came to protein, 58.8% correctly identified that chicken provides sufficient protein to support muscle growth, 35.3% of participants correctly identified that 1 cup of baked beans does not contain enough protein to promote muscle growth after resistance exercise, and 17.6% correctly identified that 1/2 cup of cooked quinoa alone is also insufficient for this purpose.

## Discussion

While previous studies have examined nutrition knowledge among adolescent athletes in other countries, limited research has been conducted in Canada. Accordingly, this study examined nutrition knowledge among adolescents aged 14–18 years participating in organized sport using the validated A-NSKQ. Overall, nutrition knowledge was low, with a mean A-NSKQ score of 47.1% correct, placing it in the poor knowledge category according to established interpretation thresholds. General nutrition knowledge scores were higher than sport nutrition knowledge scores, suggesting that adolescents were more familiar with basic nutrition concepts than with sport-specific recommendations related to performance and recovery.

### Sport nutrition knowledge gaps among adolescent athletes

The low level of nutrition knowledge observed in this study is consistent with previous research demonstrating that adolescent athletes often have inadequate understanding of both general and sport-specific nutrition concepts, including those related to energy needs, recovery nutrition, hydration, and performance nutrition (2, 3, 5). This is particularly concerning, as adolescence is a period of increased nutritional demand, during which dietary intake must simultaneously support growth, maturation, training, and training adaption (1, 2). In the present study, the mean total A-NSKQ score was below 50%, indicating poor overall knowledge and suggesting that many adolescents may lack the foundational understanding required to make informed dietary choices that support both health and sport participation (3). These findings align with Gibbs and Becker, who similarly reported that general and sport-specific nutrition knowledge scores were below 50% among adolescent athletes, highlighting persistent and substantial knowledge gaps, particularly in applied sport nutrition concepts such as fueling, recovery, hydration strategies (12).

Importantly, sport nutrition knowledge was lower than general nutrition knowledge. This finding is consistent with previous literature showing that adolescent athletes often struggle with concepts such as recovery nutrition, carbohydrate recommendations, and supplement use (2, 3, 5). Given that general nutrition messages may be more widely disseminated through school or media and sport-specific recommendations may be less accessible or less frequently reinforced, our findings may indicate that there is a need to make sport nutrition information more accessible to adolescent athletes. This may include integrating age-appropriate sport nutrition education into school- and community-based sport programs (24), increasing access to evidence-based resources developed by qualified health professionals, and providing education opportunities for coaches and parents (25, 26). Improving access to reliable sport nutrition information during adolescence may help enhance nutrition knowledge and while not examined in this study, has the potential to support healthier dietary practices aligned with training and recovery demands (27–29). However, further research is needed to clarify the extent to which improvements in knowledge translate to sustained behaviour change and long-term athletic development (28).

However, the practical relevance of specific nutrition knowledge domains may vary depending on adolescents’ level of sport participation and training demands. For example, while general nutrition concepts such as identifying fat-containing foods may reflect basic nutrition literacy, sport-specific topics such as recovery nutrition, carbohydrate fueling, and supplement use may be more directly relevant for adolescents participating in higher training volumes or competitive sport environments. Previous studies found that athletes’ knowledge is equal to or better than that of nonathletes but still significantly lower than comparison groups containing nutrition students (30). In the present study, participants reported an average of approximately 5.5 h of sport participation per week, suggesting that some adolescents may have been participating at a more recreational level. Accordingly, findings should be interpreted as reflecting nutrition knowledge among adolescents engaged in organized sport broadly, rather than exclusively trained or elite-level athletes. Therefore, not all participants may have required advanced sport nutrition knowledge to support their current activity patterns. Nonetheless, the low scores observed on several foundational sport nutrition questions (e.g., Q19: “When we exercise at a low intensity, our body mostly uses fat as a fuel”) may still indicate gaps in knowledge among adolescents who are regularly participating in organized sport and beginning to make more independent dietary decisions.

### Poor nutrition knowledge observed among both male and female athletes

No significant differences in total, general, or sport-specific nutrition knowledge scores were observed between males and females. While some studies have reported similar nutrition knowledge levels between male and female athletes, others have identified sex-based differences depending on the population studied, level of sport participation, and nutrition knowledge domains assessed (5). In studies where sex differences were observed, knowledge was greater in females that in males. Although, these differences were not consistently observed across studies and appeared to depend on varying differences of the athlete sample (30). Specifically, nutrition behaviours and variances in fruit, dairy, and sugar consumption between genders has been a notable difference in previous studies. In the present study, no significant sex differences were observed, suggesting that both male and female athletes demonstrated similarly low overall nutrition knowledge scores. Differences across studies may also reflect variations in participant age, athletic level, sample size, and nutrition knowledge assessment tools. Methodological differences may also contribute to discrepancies in findings, with previous studies highlighting the variations in quality, content, and validation of nutrition knowledge questionnaires used in athlete research (31).

### Implications for adolescent sport nutrition education

Our findings highlight potential room for improved nutrition education among Canadian adolescent athletes. Nutrition knowledge is a modifiable factor associated with dietary behaviours (7–10) and, as such, providing adolescent athletes with opportunities to learn about their sport-related nutrition needs may help support healthier dietary choices, improve nutrition practices surrounding training and recovery, and promote long-term health and athletic development. However, knowledge alone may not necessarily translate into behaviour change (9), and coaches, parents, schools, and community sport organizations should also consider environmental and behavioural factors influencing the dietary practices of adolescent athletes, such as food availability, family eating routines, time constraints, social influences, and access to reliable nutrition information (13, 14, 16). Providing greater access to sport nutrition guidance among adolescent athletes may be especially useful for athletes who compete in settings where sport dietitians, who are considered the dietary experts, are limited (7, 8). Interventions that are accessible, age-appropriate, and tailored to adolescent athletes may have the greatest likelihood of helping bridge the gap between nutrition knowledge and dietary practice (9). Involving adolescents in the development and delivery of nutrition education initiatives may also help ensure that content is relevant, engaging, and applicable to their sport participation experiences and everyday environments, thereby potentially improving the effectiveness and uptake of these interventions (13, 16).

### Strengths and limitations

Several limitations should be considered when interpreting these findings. The relatively small sample size may have limited statistical power and increased the risk of Type II error, particularly for sex-based comparisons. In addition, although participants represented multiple ethnic backgrounds, the sample was primarily composed of East Asian and European or White adolescents and may not fully reflect the broader diversity of adolescent athletes. Participants also fell within a relatively narrow age range. Furthermore, the definition of “athlete” in the present study was intentionally broad and included adolescents participating across recreational and competitive sport contexts. This may limit comparability with studies focusing specifically on elite or high-performance athlete populations. Given that participants reported an average of approximately 5.5 h of sport participation per week, some adolescents may have been participating at a more recreational level and may not have required the same level of sport-specific nutrition knowledge as highly trained or competitive athletes. Further, all respondents came from Vancouver, British Columbia, Canada. Therefore, the findings may not be generalizable to adolescent athletes with higher training volumes or performance demands. The cross-sectional design precludes causal inference; therefore, it is not possible to determine whether low nutrition knowledge leads to suboptimal dietary behaviours or whether other factors influence both knowledge and behaviour. The lack of dietary intake data further limits the ability to assess whether low knowledge translated into actual dietary practices in this sample.

All data were self-reported, which may introduce recall bias and social desirability bias (32). Adolescents may have misreported aspects of their sport participation or access to nutrition-related support, potentially influencing the accuracy of the findings. In addition, although the A-NSKQ is a validated measure of nutrition knowledge, it assesses factual knowledge rather than the ability to apply knowledge in real-world contexts (3). This is particularly relevant given that dietary behaviours in adolescents are influenced by environmental, social, and family factors (13, 14, 16).

Despite these limitations, this study has several strengths. It used a validated instrument, allowing for comparison with previous literature (3). Additionally, this study contributes novel data from a Canadian adolescent population, addressing a gap in the existing literature. Although participants represented a range of sport contexts, they shared common characteristics of regular sport participation and consistently low nutrition knowledge, suggesting that these findings may reflect a broader issue among adolescent athletes.

## Conclusion

This study explored nutrition knowledge among adolescent athletes participating in organized sport in the Vancouver area. Overall, adolescents demonstrated poor levels of nutrition knowledge and no significant differences in nutrition knowledge were observed between males and females. These findings suggest that poor nutrition knowledge may be prevalent among adolescents participating in organized sport across a broad range of recreational and competitive sport contexts in the Greater Vancouver area, irrespective of differences in training load. This may be particularly important given the limited access to professional nutrition support reported among participants, with only 6.1% indicating access to a team nutritionist or dietitian. These findings highlight a potential gap between adolescents’ nutrition needs and available resources within sport settings, and that opportunities may exist to strengthen nutrition education in adolescent sport environments. Future research using larger and more representative samples is needed to explore effective and accessible strategies for delivering nutrition education to adolescent athletes across diverse sport contexts, such as school-based sport programs, community and recreational sport clubs, and competitive youth sport environments.

## Data Availability

The raw data supporting the conclusions of this article will be made available by the authors, without undue reservation.
